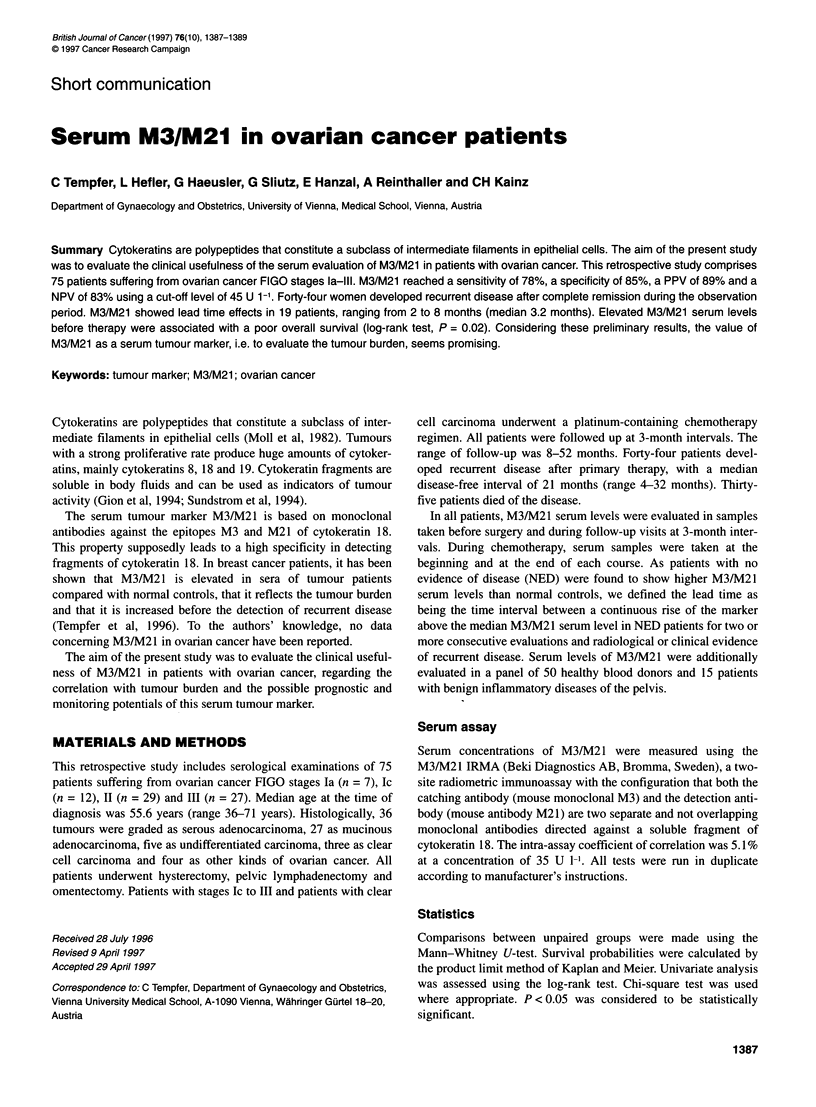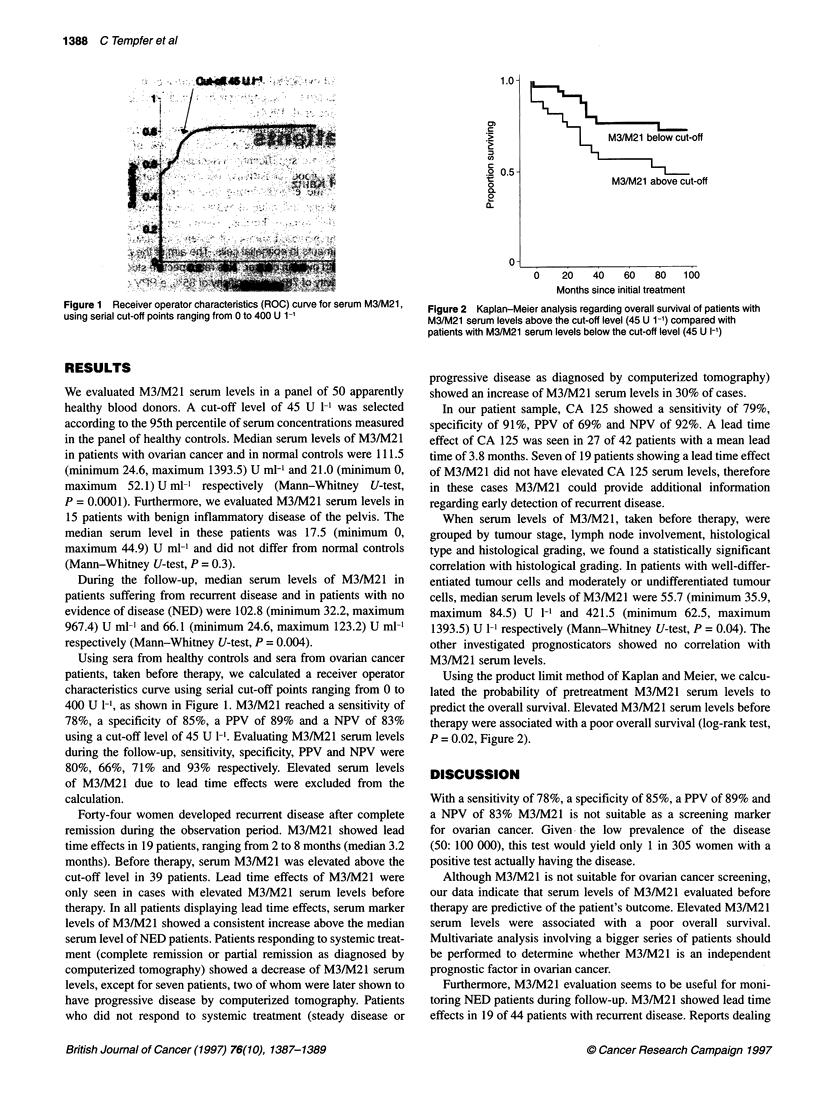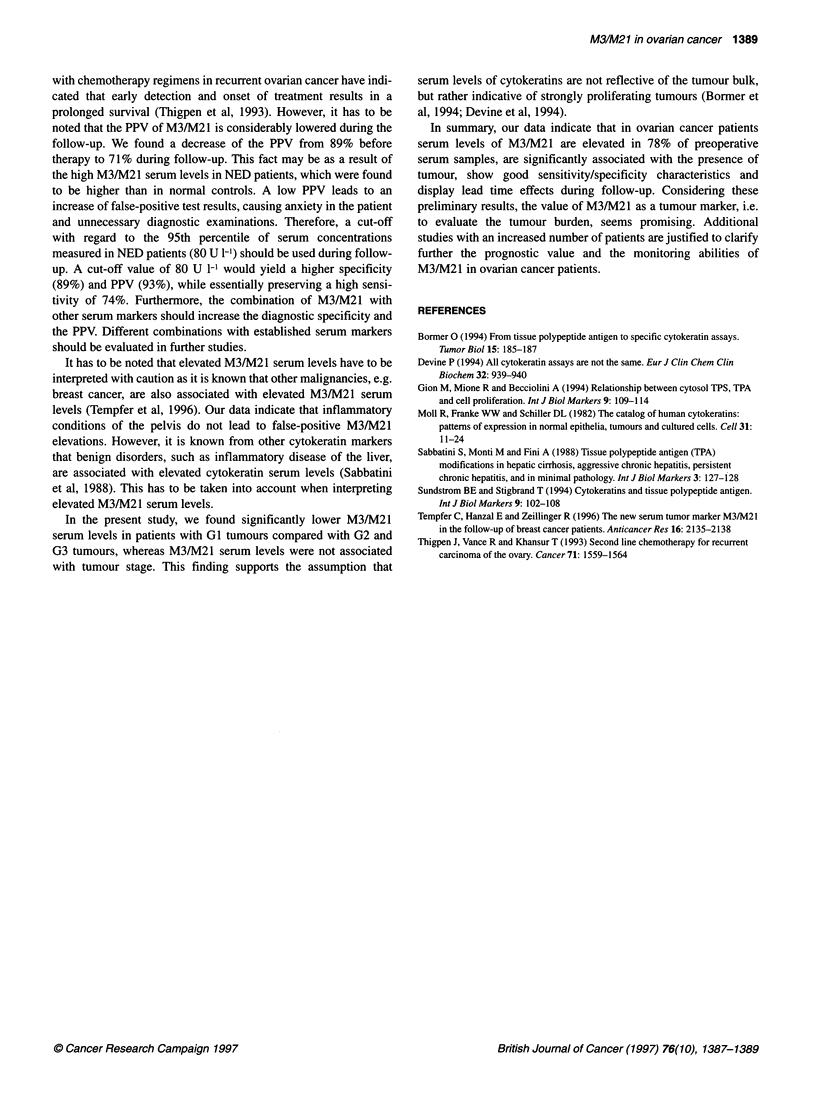# Serum M3/M21 in ovarian cancer patients.

**DOI:** 10.1038/bjc.1997.565

**Published:** 1997

**Authors:** C. Tempfer, L. Hefler, G. Haeusler, G. Sliutz, E. Hanzal, A. Reinthaller, C. H. Kainz

**Affiliations:** Department of Gynaecology and Obstetrics, University of Vienna, Medical School, Austria.

## Abstract

Cytokeratins are polypeptides that constitute a subclass of intermediate filaments in epithelial cells. The aim of the present study was to evaluate the clinical usefulness of the serum evaluation of M3/M21 in patients with ovarian cancer. This retrospective study comprises 75 patients suffering from ovarian cancer FIGO stages Ia-III. M3/M21 reached a sensitivity of 78%, a specificity of 85%, a PPV of 89% and a NPV of 83% using a cut-off level of 45 U 1(-1). Forty-four women developed recurrent disease after complete remission during the observation period. M3/M21 showed lead time effects in 19 patients, ranging from 2 to 8 months (median 3.2 months). Elevated M3/M21 serum levels before therapy were associated with a poor overall survival (log-rank test, P = 0.02). Considering these preliminary results, the value of M3/M21 as a serum tumour marker, i.e. to evaluate the tumour burden, seems promising.


*British Journal of Cancer* (1997) **76**(10), 1387–1389
© 1997 Cancer Research Campaign

Short communication

### Serum M3/M21 in ovarian cancer patients

**C Tempfer, L Hefler, G Haeusler, G Sliutz, E Hanzal, A Reinthaller and CH Kainz**

Department of Gynaecology and Obstetrics, University of Vienna, Medical School, Vienna, Austria

**Summary** Cytokeratins are polypeptides that constitute a subclass of intermediate filaments in epithelial cells. The aim of the present study was to evaluate the clinical usefulness of the serum evaluation of M3/M21 in patients with ovarian cancer. This retrospective study comprises 75 patients suffering from ovarian cancer FIGO stages Ia–III. M3/M21 reached a sensitivity of 78%, a specificity of 85%, a PPV of 89% and a NPV of 83% using a cut-off level of 45 U $1^{-1}$. Forty-four women developed recurrent disease after complete remission during the observation period. M3/M21 showed lead time effects in 19 patients, ranging from 2 to 8 months (median 3.2 months). Elevated M3/M21 serum levels before therapy were associated with a poor overall survival (log-rank test, $P$ = 0.02). Considering these preliminary results, the value of M3/M21 as a serum tumour marker, i.e. to evaluate the tumour burden, seems promising.

**Keywords:** tumour marker; M3/M21; ovarian cancer

Cytokeratins are polypeptides that constitute a subclass of intermediate filaments in epithelial cells (Moll et al, 1982). Tumours with a strong proliferative rate produce huge amounts of cytokeratins, mainly cytokeratins 8, 18 and 19. Cytokeratin fragments are soluble in body fluids and can be used as indicators of tumour activity (Gion et al, 1994; Sundstrom et al, 1994).

The serum tumour marker M3/M21 is based on monoclonal antibodies against the epitopes M3 and M21 of cytokeratin 18. This property supposedly leads to a high specificity in detecting fragments of cytokeratin 18. In breast cancer patients, it has been shown that M3/M21 is elevated in sera of tumour patients compared with normal controls, that it reflects the tumour burden and that it is increased before the detection of recurrent disease (Tempfer et al, 1996). To the authors' knowledge, no data concerning M3/M21 in ovarian cancer have been reported.

The aim of the present study was to evaluate the clinical usefulness of M3/M21 in patients with ovarian cancer, regarding the correlation with tumour burden and the possible prognostic and monitoring potentials of this serum tumour marker.

### MATERIALS AND METHODS

This retrospective study includes serological examinations of 75 patients suffering from ovarian cancer FIGO stages Ia ($n$ = 7), Ic ($n$ = 12), II ($n$ = 29) and III ($n$ = 27). Median age at the time of diagnosis was 55.6 years (range 36–71 years). Histologically, 36 tumours were graded as serous adenocarcinoma, 27 as mucinous adenocarcinoma, five as undifferentiated carcinoma, three as clear cell carcinoma and four as other kinds of ovarian cancer. All patients underwent hysterectomy, pelvic lymphadenectomy and omentectomy. Patients with stages Ic to III and patients with clear cell carcinoma underwent a platinum-containing chemotherapy regimen. All patients were followed up at 3-month intervals. The range of follow-up was 8–52 months. Forty-four patients developed recurrent disease after primary therapy, with a median disease-free interval of 21 months (range 4–32 months). Thirty-five patients died of the disease.

In all patients, M3/M21 serum levels were evaluated in samples taken before surgery and during follow-up visits at 3-month intervals. During chemotherapy, serum samples were taken at the beginning and at the end of each course. As patients with no evidence of disease (NED) were found to show higher M3/M21 serum levels than normal controls, we defined the lead time as being the time interval between a continuous rise of the marker above the median M3/M21 serum level in NED patients for two or more consecutive evaluations and radiological or clinical evidence of recurrent disease. Serum levels of M3/M21 were additionally evaluated in a panel of 50 healthy blood donors and 15 patients with benign inflammatory diseases of the pelvis.

### Serum assay

Serum concentrations of M3/M21 were measured using the M3/M21 IRMA (Beki Diagnostics AB, Bromma, Sweden), a two-site radiometric immunoassay with the configuration that both the catching antibody (mouse monoclonal M3) and the detection antibody (mouse antibody M21) are two separate and not overlapping monoclonal antibodies directed against a soluble fragment of cytokeratin 18. The intra-assay coefficient of correlation was 5.1% at a concentration of 35 U $l^{-1}$. All tests were run in duplicate according to manufacturer's instructions.

### Statistics

Comparisons between unpaired groups were made using the Mann–Whitney $U$-test. Survival probabilities were calculated by the product limit method of Kaplan and Meier. Univariate analysis was assessed using the log-rank test. Chi-square test was used where appropriate. $P < 0.05$ was considered to be statistically significant.

*Received 28 July 1996*
*Revised 9 April 1997*
*Accepted 29 April 1997*

*Correspondence to:* C Tempfer, Department of Gynaecology and Obstetrics, Vienna University Medical School, A-1090 Vienna, Währinger Gürtel 18–20, Austria

**Figure 1** Receiver operator characteristics (ROC) curve for serum M3/M21, using serial cut-off points ranging from 0 to 400 U $1^{-1}$

**Figure 2** Kaplan–Meier analysis regarding overall survival of patients with M3/M21 serum levels above the cut-off level (45 U $1^{-1}$) compared with patients with M3/M21 serum levels below the cut-off level (45 U $l^{-1}$)

### RESULTS

We evaluated M3/M21 serum levels in a panel of 50 apparently healthy blood donors. A cut-off level of 45 U $l^{-1}$ was selected according to the 95th percentile of serum concentrations measured in the panel of healthy controls. Median serum levels of M3/M21 in patients with ovarian cancer and in normal controls were 111.5 (minimum 24.6, maximum 1393.5) U $ml^{-1}$ and 21.0 (minimum 0, maximum 52.1) U $ml^{-1}$ respectively (Mann–Whitney *U*-test, $P = 0.0001$). Furthermore, we evaluated M3/M21 serum levels in 15 patients with benign inflammatory disease of the pelvis. The median serum level in these patients was 17.5 (minimum 0, maximum 44.9) U $ml^{-1}$ and did not differ from normal controls (Mann–Whitney *U*-test, $P = 0.3$).

During the follow-up, median serum levels of M3/M21 in patients suffering from recurrent disease and in patients with no evidence of disease (NED) were 102.8 (minimum 32.2, maximum 967.4) U $ml^{-1}$ and 66.1 (minimum 24.6, maximum 123.2) U $ml^{-1}$ respectively (Mann–Whitney *U*-test, $P = 0.004$).

Using sera from healthy controls and sera from ovarian cancer patients, taken before therapy, we calculated a receiver operator characteristics curve using serial cut-off points ranging from 0 to 400 U $l^{-1}$, as shown in Figure 1. M3/M21 reached a sensitivity of 78%, a specificity of 85%, a PPV of 89% and a NPV of 83% using a cut-off level of 45 U $l^{-1}$. Evaluating M3/M21 serum levels during the follow-up, sensitivity, specificity, PPV and NPV were 80%, 66%, 71% and 93% respectively. Elevated serum levels of M3/M21 due to lead time effects were excluded from the calculation.

Forty-four women developed recurrent disease after complete remission during the observation period. M3/M21 showed lead time effects in 19 patients, ranging from 2 to 8 months (median 3.2 months). Before therapy, serum M3/M21 was elevated above the cut-off level in 39 patients. Lead time effects of M3/M21 were only seen in cases with elevated M3/M21 serum levels before therapy. In all patients displaying lead time effects, serum marker levels of M3/M21 showed a consistent increase above the median serum level of NED patients. Patients responding to systemic treatment (complete remission or partial remission as diagnosed by computerized tomography) showed a decrease of M3/M21 serum levels, except for seven patients, two of whom were later shown to have progressive disease by computerized tomography. Patients who did not respond to systemic treatment (steady disease or progressive disease as diagnosed by computerized tomography) showed an increase of M3/M21 serum levels in 30% of cases.

In our patient sample, CA 125 showed a sensitivity of 79%, specificity of 91%, PPV of 69% and NPV of 92%. A lead time effect of CA 125 was seen in 27 of 42 patients with a mean lead time of 3.8 months. Seven of 19 patients showing a lead time effect of M3/M21 did not have elevated CA 125 serum levels, therefore in these cases M3/M21 could provide additional information regarding early detection of recurrent disease.

When serum levels of M3/M21, taken before therapy, were grouped by tumour stage, lymph node involvement, histological type and histological grading, we found a statistically significant correlation with histological grading. In patients with well-differentiated tumour cells and moderately or undifferentiated tumour cells, median serum levels of M3/M21 were 55.7 (minimum 35.9, maximum 84.5) U $l^{-1}$ and 421.5 (minimum 62.5, maximum 1393.5) U $l^{-1}$ respectively (Mann–Whitney *U*-test, $P = 0.04$). The other investigated prognosticators showed no correlation with M3/M21 serum levels.

Using the product limit method of Kaplan and Meier, we calculated the probability of pretreatment M3/M21 serum levels to predict the overall survival. Elevated M3/M21 serum levels before therapy were associated with a poor overall survival (log-rank test, $P = 0.02$, Figure 2).

### DISCUSSION

With a sensitivity of 78%, a specificity of 85%, a PPV of 89% and a NPV of 83% M3/M21 is not suitable as a screening marker for ovarian cancer. Given the low prevalence of the disease (50: 100 000), this test would yield only 1 in 305 women with a positive test actually having the disease.

Although M3/M21 is not suitable for ovarian cancer screening, our data indicate that serum levels of M3/M21 evaluated before therapy are predictive of the patient's outcome. Elevated M3/M21 serum levels were associated with a poor overall survival. Multivariate analysis involving a bigger series of patients should be performed to determine whether M3/M21 is an independent prognostic factor in ovarian cancer.

Furthermore, M3/M21 evaluation seems to be useful for monitoring NED patients during follow-up. M3/M21 showed lead time effects in 19 of 44 patients with recurrent disease. Reports dealing

© Cancer Research Campaign 1997

with chemotherapy regimens in recurrent ovarian cancer have indicated that early detection and onset of treatment results in a prolonged survival (Thigpen et al, 1993). However, it has to be noted that the PPV of M3/M21 is considerably lowered during the follow-up. We found a decrease of the PPV from 89% before therapy to 71% during follow-up. This fact may be as a result of the high M3/M21 serum levels in NED patients, which were found to be higher than in normal controls. A low PPV leads to an increase of false-positive test results, causing anxiety in the patient and unnecessary diagnostic examinations. Therefore, a cut-off with regard to the 95th percentile of serum concentrations measured in NED patients (80 U $l^{-1}$) should be used during follow-up. A cut-off value of 80 U $l^{-1}$ would yield a higher specificity (89%) and PPV (93%), while essentially preserving a high sensitivity of 74%. Furthermore, the combination of M3/M21 with other serum markers should increase the diagnostic specificity and the PPV. Different combinations with established serum markers should be evaluated in further studies.

It has to be noted that elevated M3/M21 serum levels have to be interpreted with caution as it is known that other malignancies, e.g. breast cancer, are also associated with elevated M3/M21 serum levels (Tempfer et al, 1996). Our data indicate that inflammatory conditions of the pelvis do not lead to false-positive M3/M21 elevations. However, it is known from other cytokeratin markers that benign disorders, such as inflammatory disease of the liver, are associated with elevated cytokeratin serum levels (Sabbatini et al, 1988). This has to be taken into account when interpreting elevated M3/M21 serum levels.

In the present study, we found significantly lower M3/M21 serum levels in patients with G1 tumours compared with G2 and G3 tumours, whereas M3/M21 serum levels were not associated with tumour stage. This finding supports the assumption that serum levels of cytokeratins are not reflective of the tumour bulk, but rather indicative of strongly proliferating tumours (Bormer et al, 1994; Devine et al, 1994).

In summary, our data indicate that in ovarian cancer patients serum levels of M3/M21 are elevated in 78% of preoperative serum samples, are significantly associated with the presence of tumour, show good sensitivity/specificity characteristics and display lead time effects during follow-up. Considering these preliminary results, the value of M3/M21 as a tumour marker, i.e. to evaluate the tumour burden, seems promising. Additional studies with an increased number of patients are justified to clarify further the prognostic value and the monitoring abilities of M3/M21 in ovarian cancer patients.

### REFERENCES

Bormer O (1994) From tissue polypeptide antigen to specific cytokeratin assays. *Tumor Biol* **15**: 185–187

Devine P (1994) All cytokeratin assays are not the same. *Eur J Clin Chem Clin Biochem* **32**: 939–940

Gion M, Mione R and Becciolini A (1994) Relationship between cytosol TPS, TPA and cell proliferation. *Int J Biol Markers* **9**: 109–114

Moll R, Franke WW and Schiller DL (1982) The catalog of human cytokeratins: patterns of expression in normal epithelia, tumours and cultured cells. *Cell* **31**: 11–24

Sabbatini S, Monti M and Fini A (1988) Tissue polypeptide antigen (TPA) modifications in hepatic cirrhosis, aggressive chronic hepatitis, persistent chronic hepatitis, and in minimal pathology. *Int J Biol Markers* **3**: 127–128

Sundstrom BE and Stigbrand T (1994) Cytokeratins and tissue polypeptide antigen. *Int J Biol Markers* **9**: 102–108

Tempfer C, Hanzal E and Zeillinger R (1996) The new serum tumor marker M3/M21 in the follow-up of breast cancer patients. *Anticancer Res* **16**: 2135–2138

Thigpen J, Vance R and Khansur T (1993) Second line chemotherapy for recurrent carcinoma of the ovary. *Cancer* **71**: 1559–1564

© Cancer Research Campaign 1997